# Real-World Patient-Reported Outcomes With Piromal in Gastroesophageal Reflux Symptoms

**DOI:** 10.14740/gr2160

**Published:** 2026-08-26

**Authors:** Marco Mantoan, Antonio Colantoni, Andrea Carlo Sironi, Valentina Nola, Elena Aragona, Giovanni Grava, Edoardo Baldini, Salvatore Emanuele Aragona

**Affiliations:** a Section of Hygiene and Preventive, Environmental and Occupational Medicine, Department of Diagnostics and Public Health, University of Verona, Verona, Italy; b Biostatistics and Data Management Consultant, Brembate, Italy; c Unit of General Surgery, ASST Melegnano Martesana, Melzo, Italy; d Department of Medicine and Surgery, University of Milano, Milan, Italy

**Keywords:** Gastroesophageal reflux, Patient-reported outcomes, Medical device, Mucosal protectant

## Abstract

**Background:**

Gastroesophageal reflux and acid-related symptoms substantially impair quality of life. Proton pump inhibitors (PPIs) remain the first-line therapy, but concerns regarding their long-term use and the persistence of symptoms in a significant proportion of patients highlight the need for complementary mucosal protection strategies. Real-world patient-reported outcomes (PROs) for barrier-forming medical devices (MDs) in individuals with reflux symptoms remain limited.

**Methods:**

An anonymous questionnaire was administered to 155 adults presenting with reflux-related symptoms. Assessments were conducted at baseline and after 7, 15, and 25 days of using the MD Piromal^®^. Symptom domains included typical reflux manifestations (heartburn, regurgitation, epigastric pain), atypical/extra-esophageal symptoms (sore throat, hoarseness, globus, throat clearing, supine cough), and dyspeptic symptoms (early satiety, postprandial fullness), rated using Likert-type severity (0–5) and frequency (0–4) scales. Nocturnal disturbances and quality-of-life impact (Verbal Numeric Rating Scale (VNRS) 0–10 for daily and social interference) were also evaluated. Changes over time were analyzed using Wilcoxon signed-rank tests. Exploratory comparisons between the MD alone (n = 133) and MD + PPI (n = 22) were performed using Wilcoxon rank-sum tests.

**Results:**

Participants had a mean age of 53.2 ± 16.7 years; 55.5% were female, and 85.8% reported predominantly typical reflux symptoms. All diurnal and nocturnal symptoms showed significant reductions at 25 days (severity/frequency P < 0.0001, except hoarseness P = 0.004), with progressive improvements observed at 7 and 15 days. Quality-of-life interference decreased markedly (daily: 7.90 ± 0.87 to 2.85 ± 1.16; social: 8.86 ± 0.89 to 2.74 ± 1.01; both P < 0.0001). Overall satisfaction was high, with 90.4% reporting outcomes exceeding expectations. The most frequently reported adverse events were nausea (23.9%) and flatulence (14.8%).

**Conclusions:**

In routine clinical practice, the use of MD was associated with substantial improvements in patient-reported upper gastrointestinal symptoms and quality-of-life measures over 25 days. Exploratory subgroup findings did not show a consistent advantage of adding PPIs across most symptom domains, suggesting that meaningful symptom relief was already observed with the MD alone in this survey. However, these findings should be interpreted as hypothesis-generating. Further controlled studies are needed to clarify the role of the MD within integrated management strategies for reflux-related symptoms.

## Introduction

Gastric hyperacidity is one of the most common gastroenterological conditions worldwide and is frequently encountered in both primary care and specialist practice [1]. It is characterized by an excessive production of hydrochloric acid, which can lead to bothersome symptoms such as heartburn, epigastric burning, and a sensation of acidity in the upper abdomen. These manifestations may significantly affect daily functioning and sleep quality, particularly when symptoms occur after meals or during the night [2].

Although often considered a transient or benign condition, persistent or inadequately controlled hyperacidity can contribute to irritation of the upper gastrointestinal mucosa. Repeated exposure to acidic gastric contents may promote inflammation and discomfort, and in some individuals may predispose to more complex clinical scenarios, including gastritis, peptic symptoms, and the early stages of gastroesophageal reflux [3]. Symptom patterns frequently fluctuate over time, with alternating periods of remission and exacerbation, making real-world assessment particularly relevant for understanding the clinical burden and the need for supportive strategies.

Gastroesophageal reflux disease (GERD) represents one of the potential evolutions of chronic acid-related symptoms. Although its pathophysiology is multifactorial and not solely dependent on acid production, gastric hyperacidity remains an important contributor to symptom generation and mucosal irritation. The increasing prevalence of GERD and its impact on quality of life (QoL) highlight the importance of early symptom management and of strategies aimed at reducing mucosal exposure to acidic content [4]. However, clinically significant overlap exists between GERD-related symptoms and functional esophageal disorders, such as functional heartburn. In this context, reducing mucosal acid exposure is mainly applicable for acid-related symptoms, whereas the evidence supporting a reliable impact on genuine functional symptoms is still limited [5, 6].

The cornerstone of the pharmacological management for acid-related disorders is represented by proton pump inhibitors (PPIs), which are highly effective in reducing gastric acid secretion. However, despite their widespread use, many patients continue to experience residual symptoms such as postprandial discomfort, nocturnal acidity, or extra-esophageal manifestations. These persistent symptoms may reflect the multifactorial nature of acid-related conditions, in which mucosal protection, esophageal clearance, and mechanical factors contribute to symptom generation independently of acid suppression. In addition, concerns have arisen about the improper or prolonged use of PPIs, especially in patients without a clear indication or those who continue therapy beyond the recommended duration [7]. Current clinical guidelines highlight the importance of periodic reassessment, the use of the lowest effective dose, and the consideration of non-pharmacological strategies when appropriate [8]. This has increased interest in supportive approaches that can complement pharmacological therapy or provide symptom relief in individuals with mild, intermittent, or fluctuating acid-related symptoms [9].

Therefore, non-pharmacological interventions have been receiving increasing attention to reduce mucosal exposure to acidic gastric contents and enhance mucosal protection, particularly due to the increasing emphasis on deprescribing strategies [10]. The variety of symptoms experienced by patients with hyperacidity over time necessitates the availability of safe options for daily therapy, which can also be employed during symptom recurrence or while waiting for diagnostic evaluation.

Within this context, real-world data are crucial to comprehend how supportive strategies are perceived and used in routine clinical practice, and how they may contribute to symptom control alongside or independently of pharmacological treatment [11].

The management of acid-related symptoms increasingly recognizes the importance of strategies that reduce mucosal exposure to acidic gastric contents and support the integrity of the upper gastrointestinal barrier. While pharmacological agents such as PPIs act by suppressing acid secretion, non-pharmacological approaches can provide complementary benefits by limiting direct contact between the mucosa and refluxed or acidic material [12]. The barrier-forming and mucosal-protective medical devices (MDs) are designed to act locally within the upper gastrointestinal tract, producing a transient protective layer that can assist in reducing irritation and discomfort related to hyperacidity [13]. These formulations typically combine components with different but synergistic functions, such as agents capable of forming a physical barrier, substances that adhere to the mucosal surface, and compounds with buffering capacity that can transiently reduce the acidity of gastric contents. Through these mechanisms, such devices may contribute to symptom relief by decreasing mucosal stress and improving the perception of upper gastrointestinal comfort. In order to assess the validity of this rationale, this article evaluates the effects of the Piromal® (Tamaracid® plus; manufacturer S.I.I.T. srl, Trezzano s/N, Milan, Italy) MD. Its formulation includes sodium alginate, a polysaccharide that rapidly forms a viscous gel in the presence of gastric acid and contributes to creating a physical barrier that limits upward movement of gastric contents; calcium carbonate and potassium bicarbonate, which provide a mild and transient buffering effect; and *Tamarindus indica* seed extract, a plant-derived polysaccharide with documented mucoadhesive and film-forming properties that supports the protective function of the upper gastrointestinal mucosa [14]. This combination is intended to act locally without systemic absorption, offering a supportive option for individuals experiencing acid-related symptoms, either alone or alongside standard pharmacological therapy, depending on clinical needs and guideline-based decision-making [15].

A study examined prescribing habits among Italian cardiologists, revealing a marked preference for PPIs over alginates and mucosal protectants, despite increasing concerns regarding the long-term use of PPIs in patients without a clear indication [16]. The study also highlighted that positive clinical experiences with mucosal-protective MDs were generally well-considered and could influence therapeutic decision-making.

Despite these contributions, important knowledge gaps remain. Real-world data on the use of this MD in everyday clinical practice are limited, particularly regarding symptom patterns, perceived effectiveness, nocturnal and atypical symptoms, and the integration of mucosal-protective strategies with or without pharmacological therapy. Understanding how patients experience and evaluate such devices in routine settings is essential to better characterize their role in the management of acid-related symptoms.

Given the high prevalence of acid-related symptoms, the limitations of current pharmacological strategies, and the growing interest in non-pharmacological approaches that protect the upper gastrointestinal mucosa, additional real-world evidence is necessary to better define the usage and perception of mucosal-protective MDs in everyday clinical practice. However, data remain limited regarding symptom patterns, perceived benefits, nocturnal and atypical manifestations, and the integration of such devices with or without concomitant pharmacological therapy [17].

GERD symptoms encompass typical manifestations (heartburn, regurgitation, epigastric pain), atypical (sore throat, hoarseness, globus sensation, cough worsening supine), and overlapping dyspeptic features (early satiety, postprandial fullness), often uncorrelated with endoscopic findings. Nocturnal symptoms and QoL impairment are frequent, best captured by validated patient-reported outcome (PRO) instruments such as Gastroesophageal Reflux Disease Questionnaire (GERD-Q), Gastroesophageal Symptom Assessment Scale (GSAS), or Reflux Disease Questionnaire (RDQ), though simple Likert and Verbal Numeric Rating Scale (VNRS) scales enable reproducible real-world assessment. This survey evaluated these symptom domains to characterize burden and patient experience with Piromal^®^ in routine practice.

The aim of this survey was therefore to evaluate the real-world experience of individuals using the MD, assessing changes in typical, atypical, dyspeptic, and nocturnal symptoms, as well as perceived effectiveness, tolerability, and overall impact on upper gastrointestinal comfort. By exploring PROs in routine settings, this study contributes to a more comprehensive understanding of the role of mucosal-protective MD in the management of acid-related symptoms.

## Materials and Methods

The study was conducted in accordance with the Declaration of Helsinki. According to local institutional policy for fully anonymized, non-interventional surveys, formal approval by an ethics committee was not required. In accordance with European Union (EU) Regulation 2016/679 (General Data Protection Regulation (GDPR)), recital 26, all data were collected in fully anonymized form, without any possibility of re-identification. No personal identifiers (e.g., name, date of birth, contact information, clinical record number) were collected or stored. The questionnaire was administered by gastroenterologists during routine clinical practice, but responses were recorded in an anonymous format and could not be linked to individual patients. As data were fully anonymized and no identifiable personal information was recorded, individual written informed consent was not required. Questionnaires were anonymously administered by gastroenterologists to 155 patients during routine visits to evaluate the effectiveness, safety, and usability of the MD. From each subject, anonymized information on gender, age, prevalent symptom type (typical, atypical, or both), and concomitant use of PPI was collected.

### Questionnaire design

The questionnaire, specifically designed for this real-world survey, was not subjected to formal validation. Its structure was based on established domains commonly used in validated GERD instruments, such as symptom frequency, severity, and nocturnal disturbances, as previously described in the literature. In particular, the rating approach was adapted from elements of the RDQ and the GERD Impact Scale, while the overall treatment evaluation format was modeled on prior survey-based approaches reported in the literature [18, 19].

The questionnaire was formulated based on input from gastroenterologists in everyday clinical practice to represent a pragmatic, real-world perspective for symptom assessment and to ensure feasibility within routine clinical settings.

Although this simplified instrument was considered suitable for exploratory use in routine care, its results cannot be directly compared with those obtained using fully validated questionnaires.

Four time points were considered: baseline (72 h before treatment), 7 days (T1), 15 days (T2), and 25 days (T3) after treatment. Symptoms of gastroesophageal reflux were categorized into three groups: typical, atypical, and dyspeptic. Typical symptoms included heartburn, acid regurgitation, and epigastric pain. Atypical symptoms included sore throat, hoarseness, sensation of a “knot” in the throat, throat clearing, and persistent cough worsening in the supine position. Dyspeptic symptoms comprised early satiety, postprandial fullness, and epigastric burning or pain.

For each of these symptoms, both severity and frequency were evaluated. Severity was rated on a 6-point Likert scale (0 = absence of symptoms; 5 = very severe), while frequency was rated on a 5-point Likert scale (0 = never; 4 = every day).

Nocturnal symptoms were also evaluated. The frequency of sleep disturbances due to symptoms was rated on a 4-point scale (0 = never; 3 = every day). For cases reporting a score of ≥ 1, the severity of typical, atypical, and dyspeptic symptoms was additionally rated.

The impact of symptoms on daily and social activities was investigated using a VNRS ranging from 0 (“no perception”) to 10 (“worst possible perception”) to evaluate their impact on the individual’s QoL.

The overall perception of symptom improvement was evaluated using a 4-point Likert scale ranging from –1 (“worsening”) to +2 (“high improvement”). Perceived effectiveness of the product on typical symptoms, atypical symptoms, and gastric hyperacidity was rated on a 5-point Likert scale (“excellent” to “poor”). Patient expectations were categorized from “much better than expected” to “worse than expected”.

Safety monitoring included the evaluation of side effects, palatability, tolerability, ease of use, and overall satisfaction. Side effects were collected as dichotomous PROs at the end of follow-up, referring to symptoms experienced during treatment, whereas palatability, tolerability, ease of use, and overall satisfaction were rated on a 5-point Likert scale. Since the questionnaire was administered at the end of follow-up through questions referring to symptoms experienced during treatment, no baseline assessment or severity grading was available for these events.

### Study population

Participants were selected among patients who had already received a clinical diagnosis of GERD from their treating specialist and were using the MD in routine practice. In cases of diagnostic uncertainty, the treating specialist could request further evaluation, including endoscopy. However, this diagnostic process occurred before the selection of cases for the study and was not part of the present survey. Since the data were collected anonymously, individual diagnostic details could not be retrieved.

### Statistical analysis

Quantitative variables were summarized using the number of observations, mean, standard deviation, median, and range (minimum–maximum). Categorical variables were summarized as absolute frequencies and percentages. Changes from baseline to the end of treatment (T3, 25 days) were evaluated using the Wilcoxon signed-rank test for paired data. Secondary analyses comparing baseline with T1 (7 days) and T2 (15 days) were performed using the same approach. A subgroup comparison between subjects treated with the MD alone and those treated with the MD plus PPIs was conducted using the Wilcoxon rank-sum test for independent samples. Given the exploratory nature of the study and the descriptive purpose of the survey, no correction for multiple comparisons was applied. Statistical significance was set at a two-tailed P value < 0.05. All analyses were performed using SAS software, version 9.4 (SAS Institute Inc., Cary, NC, USA).

## Results

### Participant characteristics

A total of 155 subjects were included in the study. The mean age was 53.24 ± 16.7 years (range 19–93), and 55.5% were female (86/155), while 44.5% were male (69/155). Most participants reported predominantly typical GERD symptoms, whereas 16.8% presented a combination of typical and atypical symptoms.

Regarding concomitant therapies, 133 subjects (85.8%) used MD alone, while 22 subjects (14.2%) used it in combination with a PPI. The distribution of demographic and clinical characteristics is summarized in Table 1.

**Table 1:** Participant Demographics and Clinical Characteristics (N = 155)

Characteristic	N (%)
Gender	
Male	69 (44.5%)
Female	86 (55.5%)
Age (years)	
Mean (SD)	53.24 (16.69)
Median (minimum–maximum)	55 (19.00–93.00)
Diagnosis of GERD (symptoms)	
Typical (gastro-esophageal)	129 (83.2%)
Typical/atypical (gastro-esophageal/laryngo-pharyngeal)	26 (16.8%)
Concomitant use of PPI	
No	133 (85.8%)
Yes	22 (14.2%)

SD: standard deviation; GERD: gastroesophageal reflux disease; PPIs: proton pump inhibitors.

### Primary outcomes

**Figure 1:**
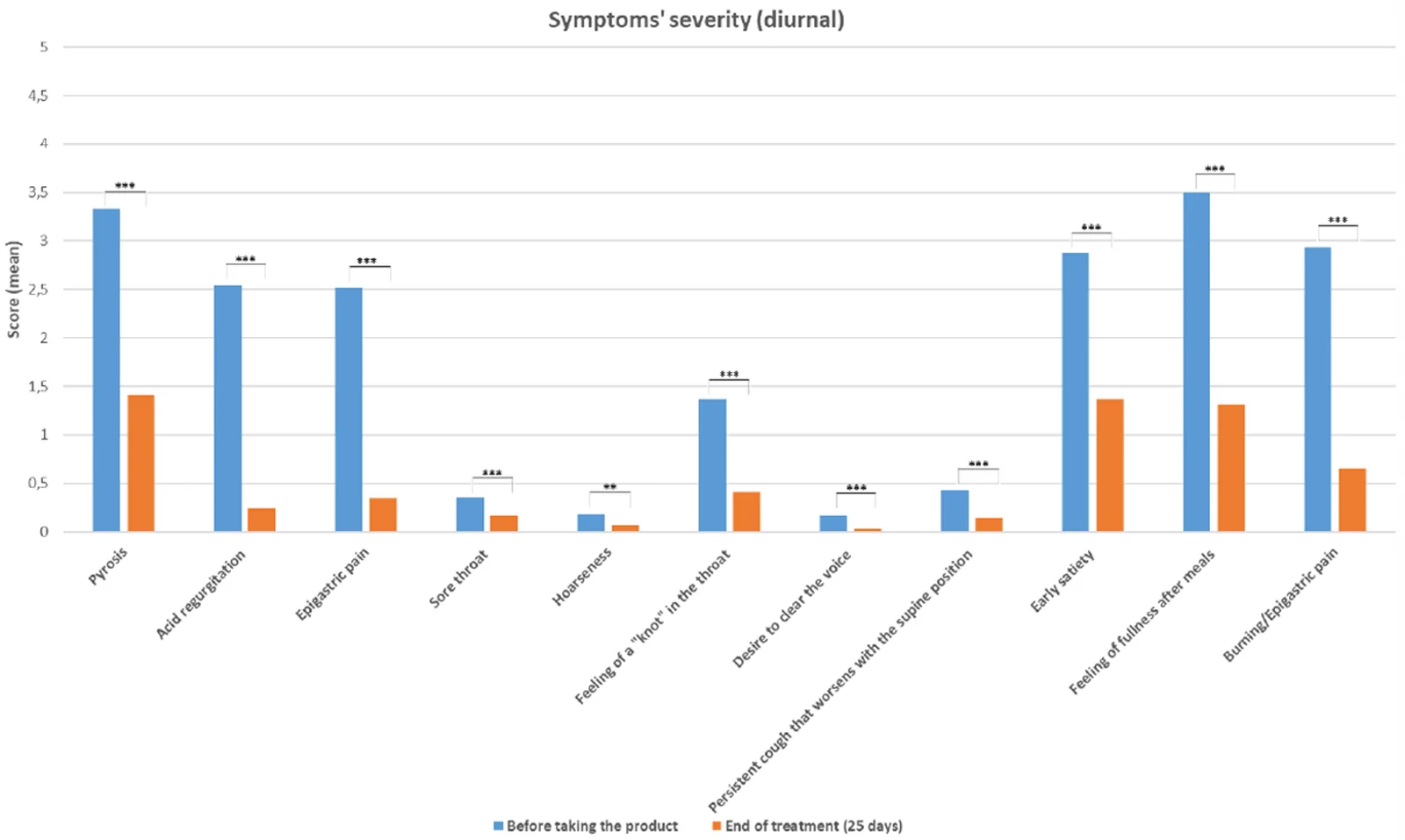
Change in symptom severity score from baseline to T3 (25 days). ***P < 0.0001; hoarseness **P = 0.0004.

**Figure 2:**
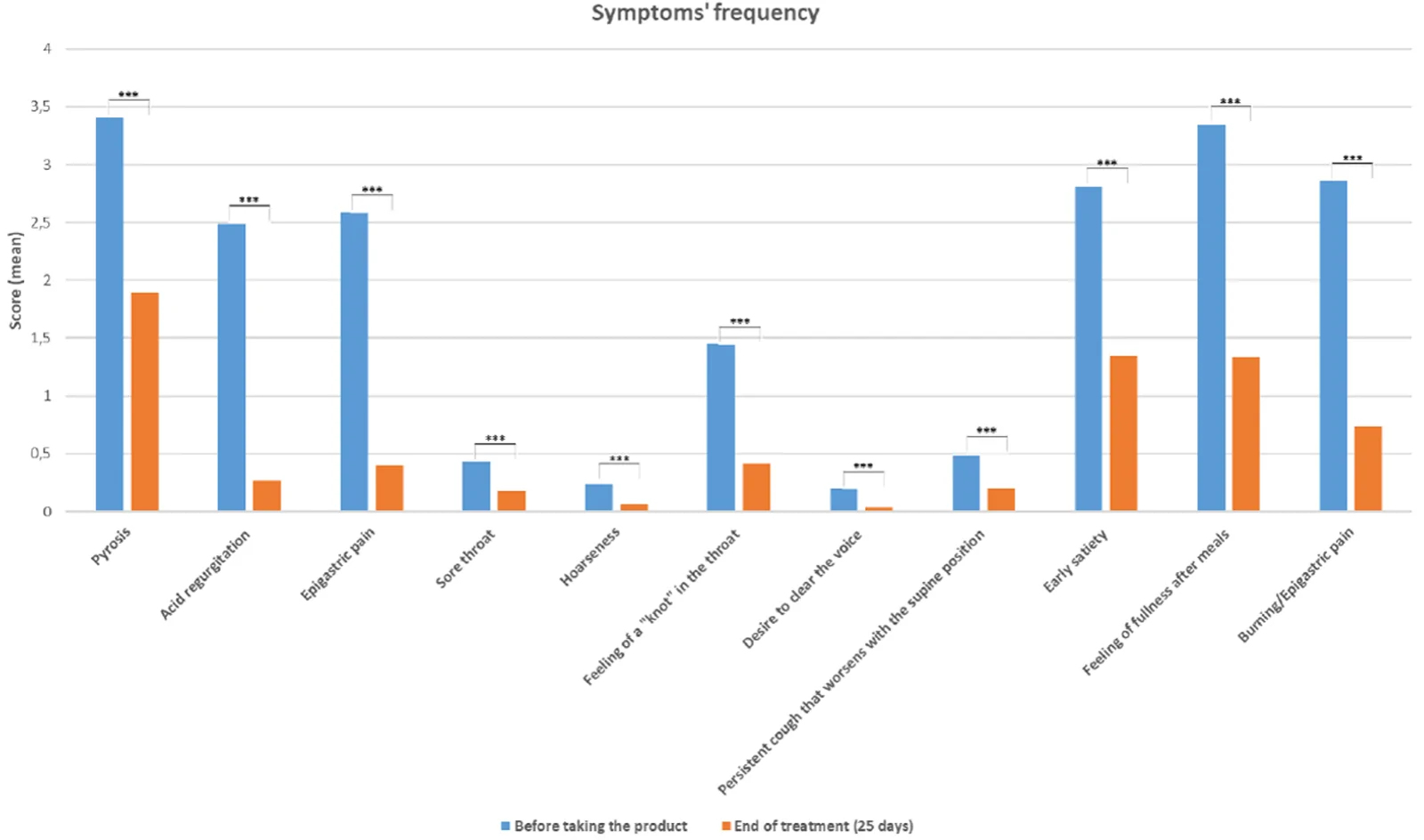
Change in symptom frequency score from baseline to T3 (25 days). ***P < 0.0001.

A significant improvement was observed across all diurnal symptoms. Both severity and frequency scores decreased substantially from baseline to T3 (25 days), with all comparisons reaching high statistical significance (P < 0.0001) for all parameters, except for hoarseness (P = 0.004). Mean changes for each symptom are reported in Tables 2, and 3, while Figures 1 and 2 illustrate the overall reduction patterns.

**Table 2:** Change in Severity Score of Symptoms From Baseline to T3 (25 Days)

Symptom	Value at baseline (mean ± SD)	Value at T3 (mean ± SD)	Mean difference ± SD	P value
Pyrosis	3.33 ± 0.72	1.41 ± 0.67	–1.92 ± 0.78	< 0.0001
Acid regurgitation	2.54 ± 0.90	0.24 ± 0.50	–2.30 ± 0.85	< 0.0001
Epigastric pain	2.52 ± 0.88	0.35 ± 0.53	–2.17 ± 0.79	< 0.0001
Sore throat	0.36 ± 0.67	0.17 ± 0.39	–0.19 ± 0.47	< 0.0001
Hoarseness	0.18 ± 0.46	0.07 ± 0.28	–0.11 ± 0.39	0.004
Feeling of a “knot” in the throat	1.37 ± 1.24	0.41 ± 0.61	–0.97 ± 0.98	< 0.0001
Desire to clear the voice	0.17 ± 0.47	0.03 ± 0.21	–0.14 ± 0.45	< 0.0001
Persistent cough that worsens with the supine position	0.43 ± 0.72	0.15 ± 0.40	–0.28 ± 0.56	< 0.0001
Early satiety	2.88 ± 0.75	1.37 ± 0.76	–1.51 ± 0.66	< 0.0001
Feeling of fullness after meals	3.50 ± 0.77	1.32 ± 0.76	–2.19 ± 0.72	< 0.0001
Burning/epigastric pain	2.93 ± 0.83	0.66 ± 0.66	–2.26 ± 0.69	< 0.0001

SD: standard deviation.

**Table 3:** Change in Frequency Score of Symptoms From Baseline to T3 (25 Days)

Symptom	Value at baseline (mean ± SD)	Value at T3 (mean ± SD)	Mean difference ± SD	P value
Pyrosis	3.41 ± 0.72	1.89 ± 0.83	–1.52 ± 0.79	< 0.0001
Acid regurgitation	2.49 ± 0.92	0.27 ± 0.54	–2.22 ± 0.85	< 0.0001
Epigastric pain	2.59 ± 0.86	0.40 ± 0.62	–2.19 ± 0.78	< 0.0001
Sore throat	0.43 ± 0.88	0.18 ± 0.42	–0.25 ± 0.66	< 0.0001
Hoarseness	0.24 ± 0.59	0.06 ± 0.27	–0.17 ± 0.54	< 0.0001
Feeling of a “knot” in the throat	1.45 ± 1.34	0.42 ± 0.64	–1.03 ± 1.10	< 0.0001
Desire to clear the voice	0.20 ± 0.53	0.04 ± 0.22	–0.16 ± 0.55	< 0.0001
Persistent cough that worsens with the supine position	0.48 ± 0.84	0.20 ± 0.50	–0.28 ± 0.60	< 0.0001
Early satiety	2.81 ± 0.77	1.35 ± 0.76	–1.46 ± 0.79	< 0.0001
Feeling of fullness after meals	3.34 ± 0.82	1.33 ± 0.88	–2.01 ± 0.82	< 0.0001
Burning/epigastric pain	2.86 ± 0.89	0.74 ± 0.75	–2.13 ± 0.81	< 0.0001

SD: standard deviation.

**Figure 3:**
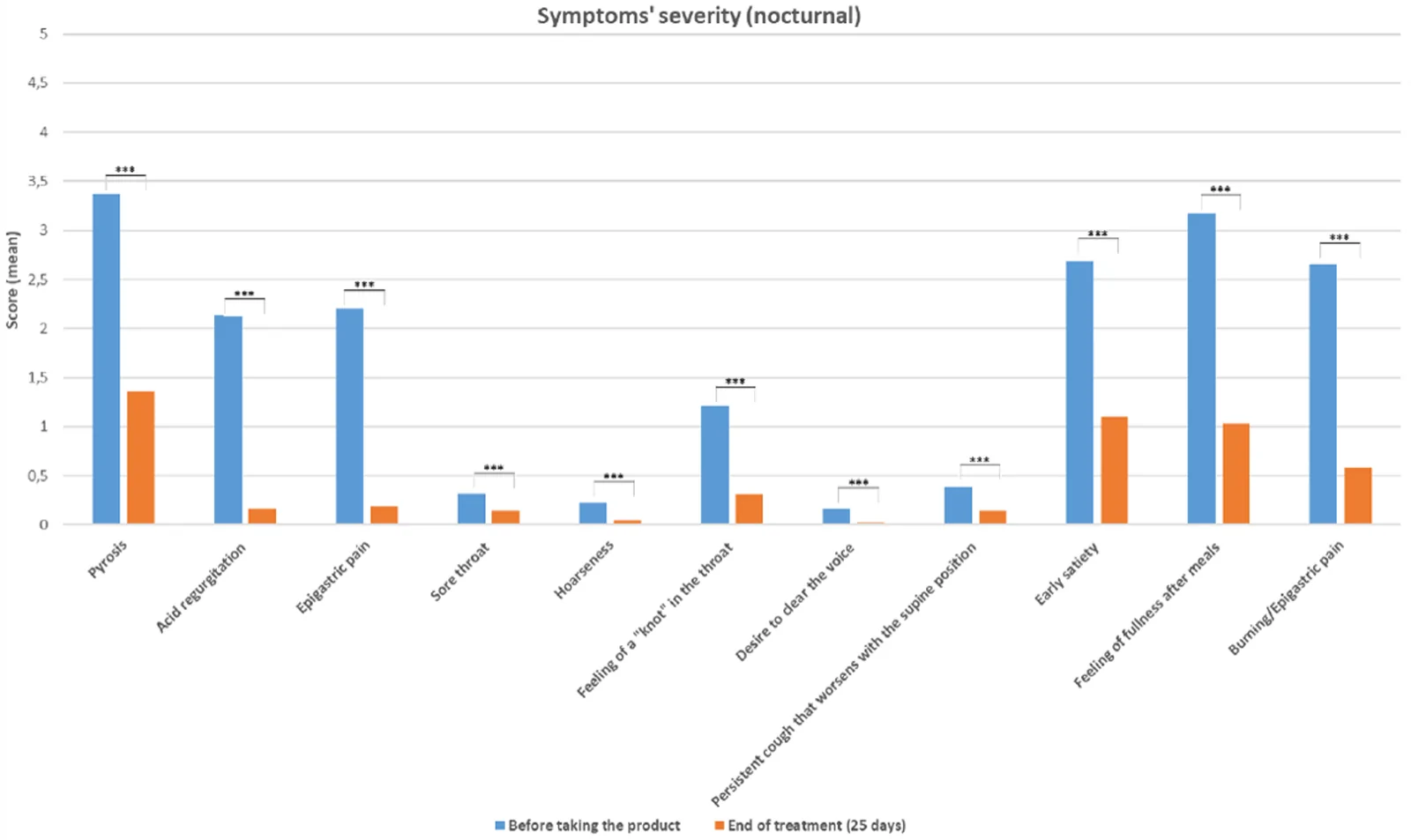
Change in nocturnal symptom scores from baseline to T3 (25 days). ***P < 0.0001.

Nocturnal symptoms also improved markedly. The mean frequency of sleep disturbances decreased from 2.77 ± 0.44 at baseline to 1.10 ± 0.36 at T3 (P < 0.0001). Severity scores for nocturnal symptoms showed similar reductions, as detailed in Table 4 and illustrated in Figure 3.

**Table 4:** Change in Nocturnal Symptom Scores From Baseline to T3 (25 Days)

Symptom	Value at baseline (mean ± SD)	Value at T3 (mean ± SD)	Mean difference ± SD	P value
Pyrosis	3.37 ± 0.71	1.36 ± 0.64	–2.01 ± 0.74	< 0.0001
Acid regurgitation	2.13 ± 1.04	0.16 ± 0.89	–1.97 ± 0.89	< 0.0001
Epigastric pain	2.20 ± 0.98	0.19 ± 0.43	–2.01 ± 0.93	< 0.0001
Sore throat	0.32 ± 0.69	0.14 ± 0.43	–0.18 ± 0.53	< 0.0001
Hoarseness	0.22 ± 0.56	0.05 ± 0.21	–0.17 ± 0.50	< 0.0001
Feeling of a “knot” in the throat	1.21 ± 1.24	0.31 ± 0.56	–0.90 ± 0.98	< 0.0001
Desire to clear the voice	0.16 ± 0.45	0.02 ± 0.18	–0.14 ± 0.48	< 0.0001
Persistent cough that worsens with the supine position	0.39 ± 0.70	0.14 ± 0.38	–0.26 ± 0.52	< 0.0001
Early satiety	2.68 ± 0.82	1.10 ± 0.71	–1.59 ± 0.76	< 0.0001
Feeling of fullness after meals	3.17 ± 0.88	1.03 ± 0.74	–2.14 ± 0.85	< 0.0001
Burning/epigastric pain	2.66 ± 0.94	0.58 ± 0.62	–2.08 ± 0.88	< 0.0001

SD: standard deviation.

**Figure 4:**
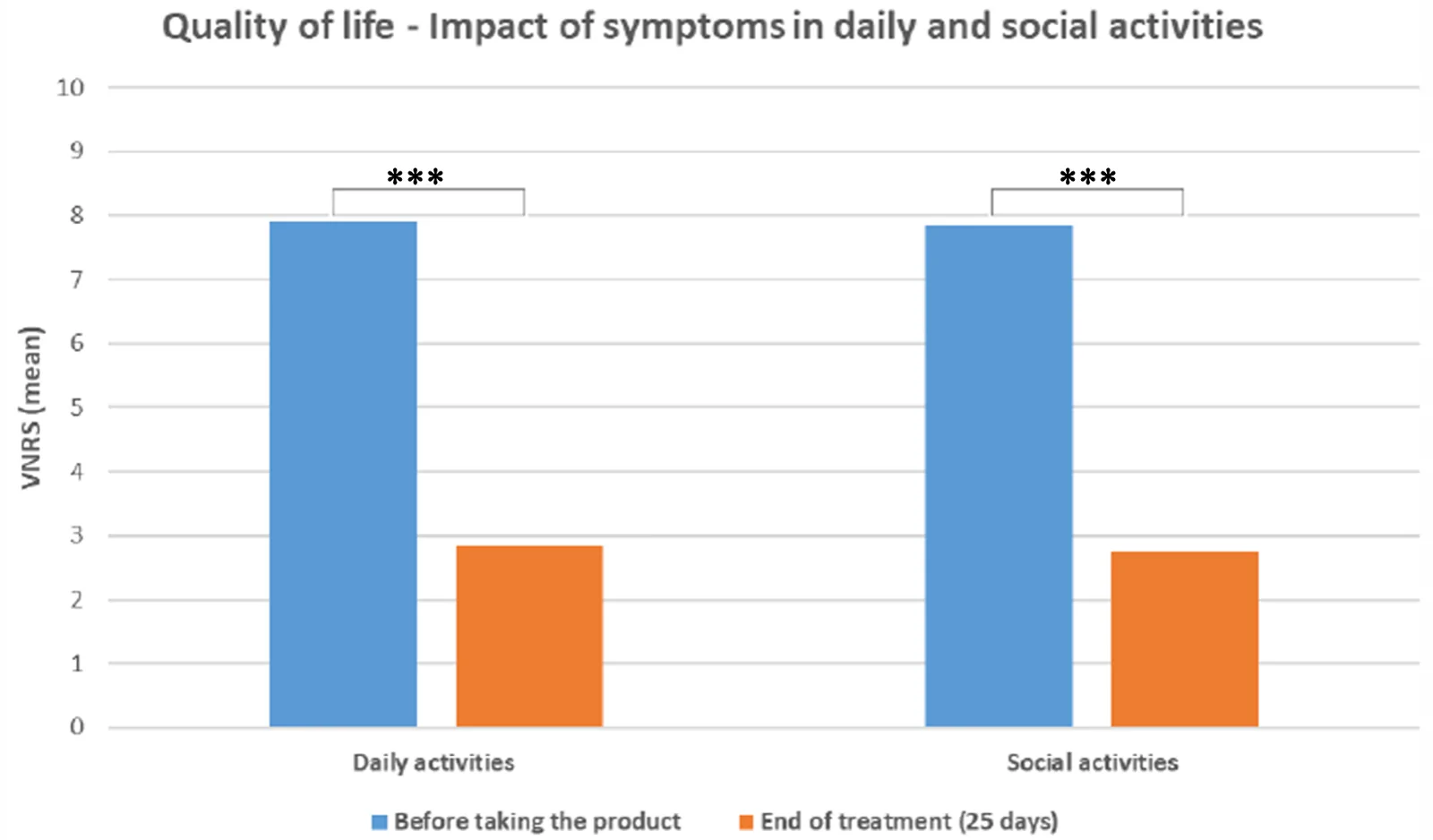
Change in VNRS quality-of-life scores from baseline to T3 (25 days). ***P < 0.0001. VNRS: Verbal Numeric Rating Scale.

QoL improved significantly. VNRS scores decreased from baseline (daily: 7.90 ± 0.87; social: 8.86 ± 0.89) to day 25 (daily: 2.85 ± 1.16; social: 2.74 ± 1.01; both P < 0.0001; Fig. 4).

### Secondary outcome

**Figure 5:**
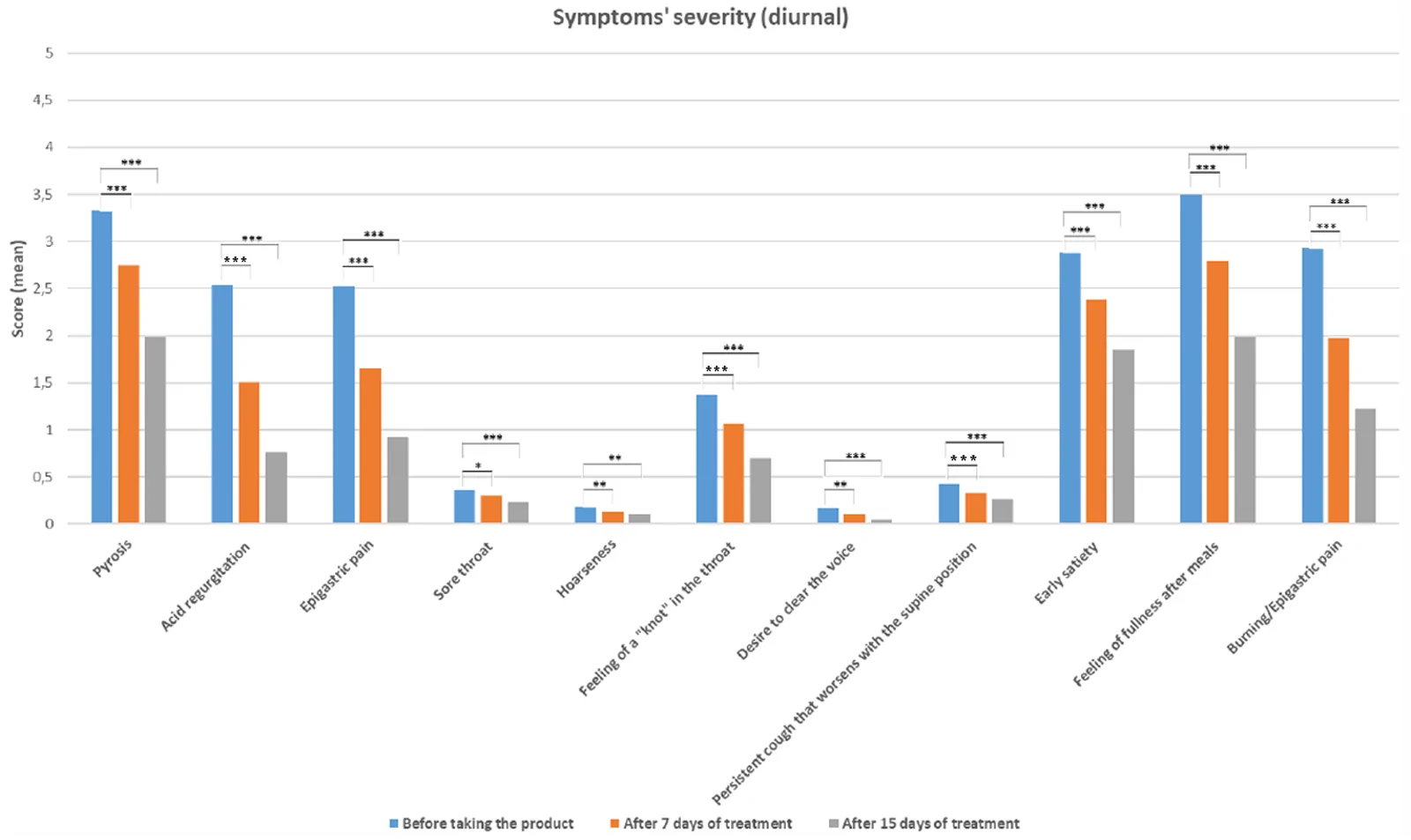
Change in symptom severity score at T1 (7 days) and T2 (15 days). *P < 0.05. **P < 0.01. ***P < 0.0001.

**Figure 6:**
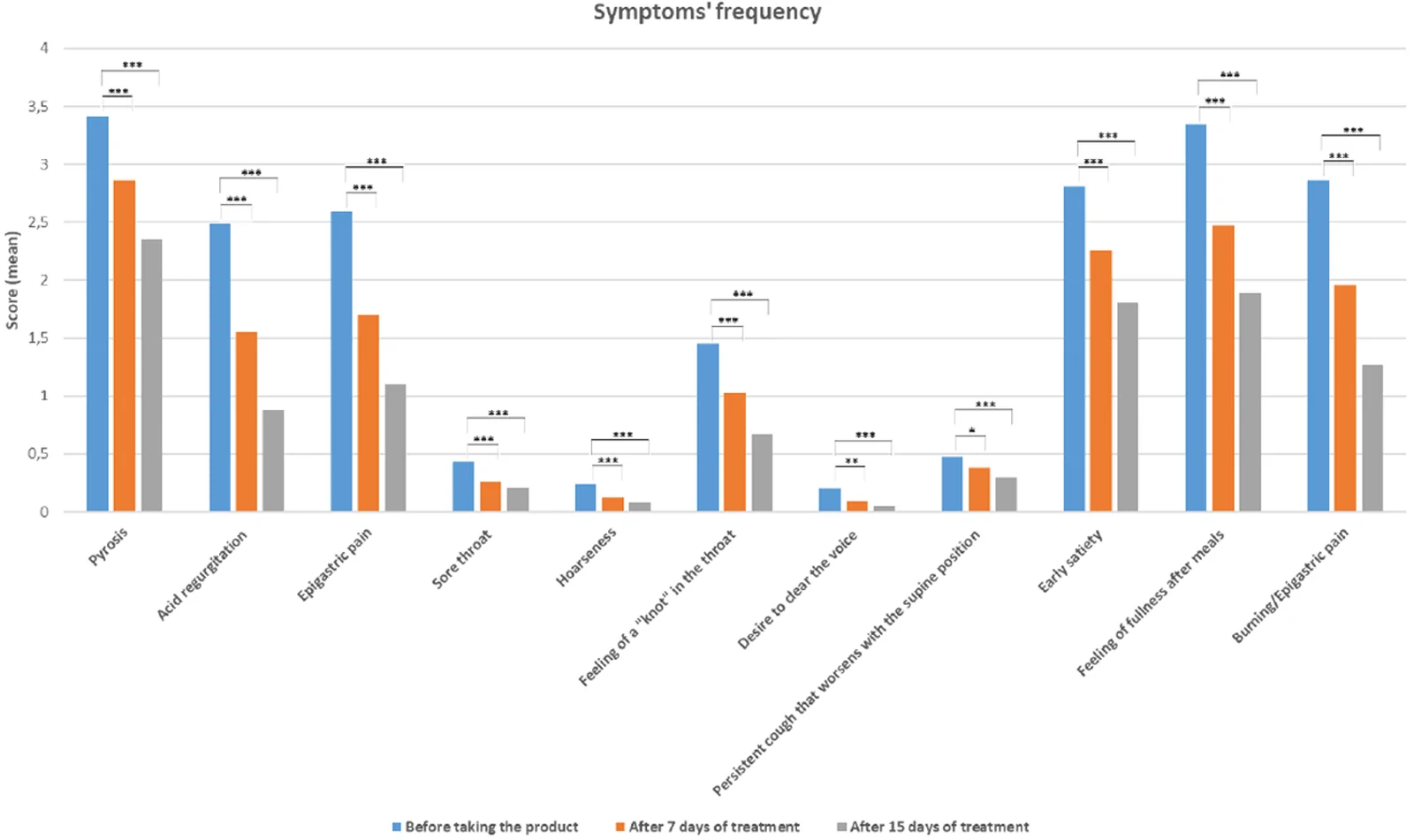
Change in symptom frequency score at T1 (7 days) and T2 (15 days). *P < 0.05. **P < 0.01. ***P < 0.0001.

Progressive improvements were observed at both T1 (7 days) and T2 (15 days) across all symptom categories. Diurnal symptom severity decreased significantly at T1 and continued to improve at T2, with consistent reductions across typical, atypical, and dyspeptic symptoms (Fig. 5). Frequency scores showed a similar pattern, with early improvement at T1 and more reductions by T2 (Fig. 6).

**Figure 7:**
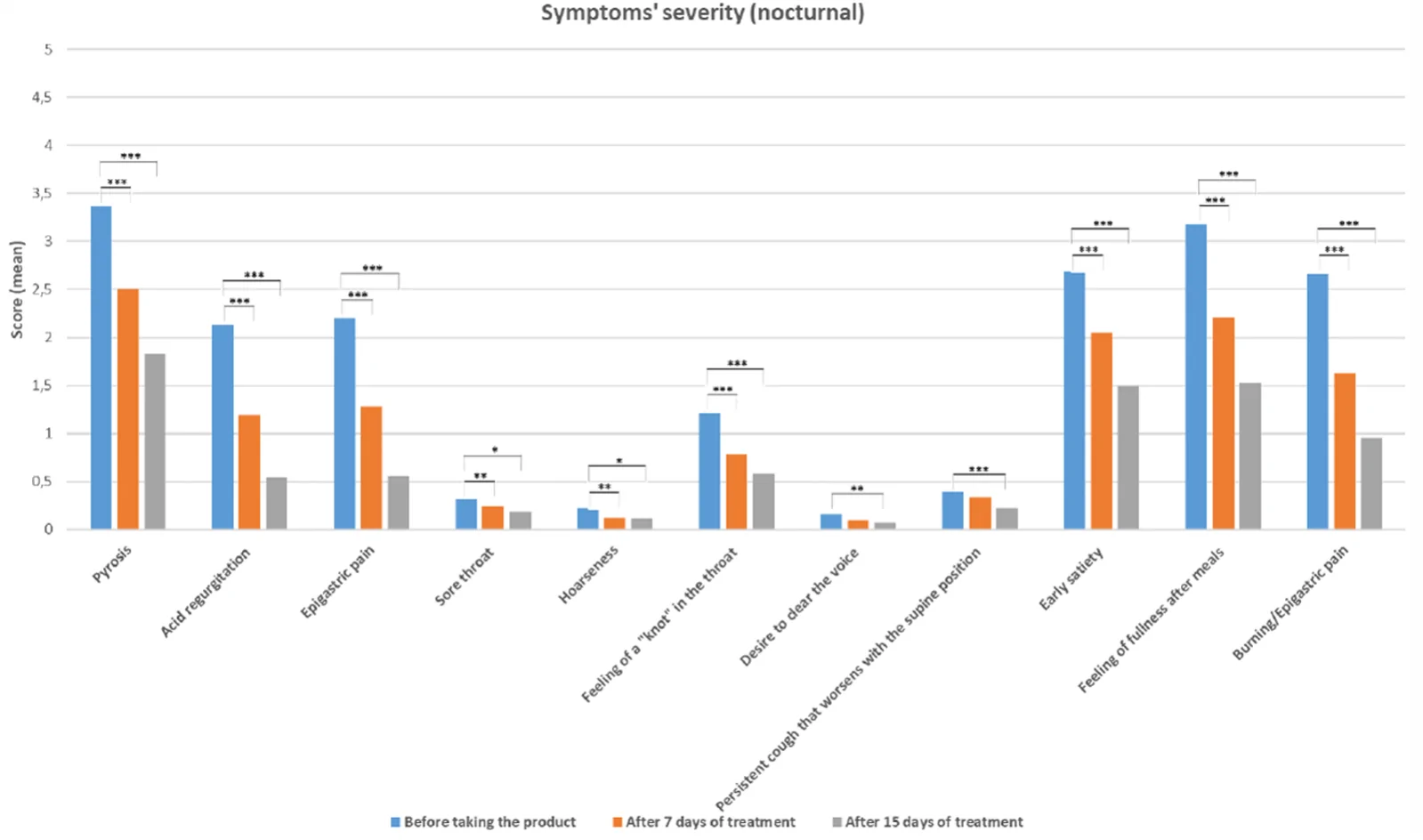
Change in nocturnal symptom scores at T1 (7 days) and T2 (15 days). *P < 0.05. **P < 0.01. ***P < 0.0001.

**Figure 8:**
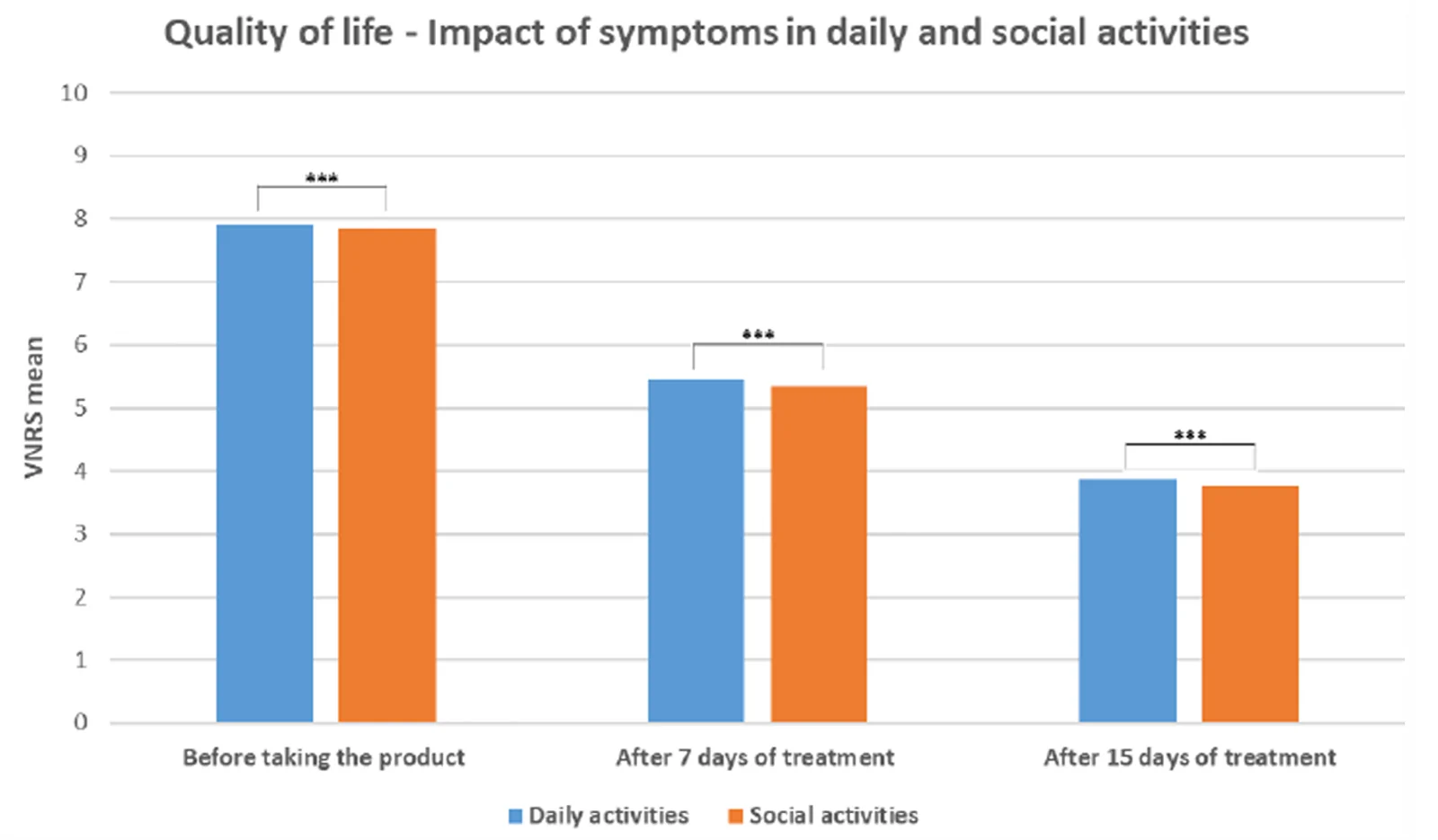
Change in VNRS quality-of-life scores at T1 (7 days) and T2 (15 days). ***P < 0.0001.

**Figure 9:**
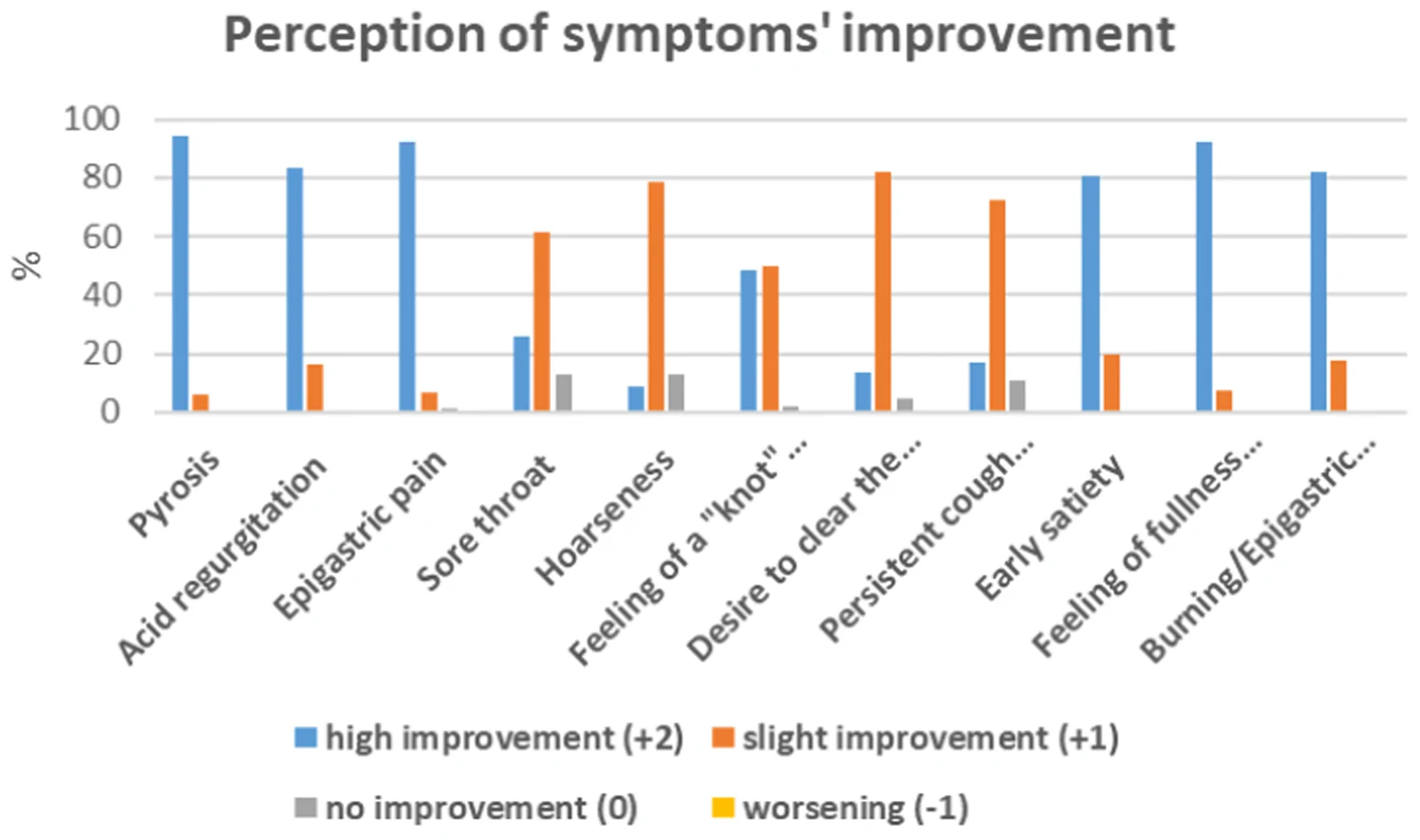
User-reported perceived improvement for each symptom.

Nocturnal symptoms also improved progressively. Severity scores decreased significantly at T1 and showed additional improvement at T2 (Fig. 7). Similarly, the QoL measures reflected these trends: VNRS scores for interference with daily and social activities decreased significantly at both times, with further improvements at T2 (Fig. 8). Perceived improvement for each symptom is summarized in Figure 9. Perceived effectiveness was predominantly rated in the higher categories for typical, atypical, and dyspeptic symptoms.

### Safety and tolerability

Overall acceptability was favorable. Most participants did not report adverse effects. The most frequently reported treatment-emergent events were nausea (23.9%) and flatulence (14.85%), followed by constipation (1.3%) and vomiting (0.6%), while no cases of diarrhea, headache, or other adverse events were recorded. These events were collected as dichotomous PROs at study completion and were not graded for severity. Ratings for palatability, tolerability, and ease of use were generally in the upper range of the 5-point Likert scale.

### Subgroup analysis: MD vs MD + PPI

Of 155 participants, 133 (85.8%) used MD alone and 22 (14.2%) used MD + PPI. Both groups showed substantial symptom reductions from baseline to T3. Exploratory between-group comparisons revealed numerically greater improvements in select symptoms for MD + PPI (Tables 5, 6). Statistically significant differences (P < 0.05) were limited to pyrosis severity (P = 0.0146), selected atypical symptoms, and nocturnal sleep disturbance frequency (P = 0.0240). Interpretation was limited by the small PPI subgroup (n = 22), baseline imbalances, and absence of randomization. These subgroup comparisons should therefore be interpreted as exploratory only, as they were underpowered and were not adjusted for multiple testing or confounders.

**Table 5:** Change in Symptom Severity Score From Baseline to T3 in the MD Only and MD + PPI Groups

Symptom	Variations at T3 vs. baseline (mean ± SD)/MD ± PPI	Variations at T3 vs. baseline (mean ± SD)/MD only	P value
Pyrosis	–2.27 ± 0.63	–1.86 ± 0.79	0.0146*
Acid regurgitation	–2.14 ± 1.04	–2.32 ± 0.82	0.0266
Epigastric pain	–2.23 ± 0.81	–2.16 ± 0.79	0.6459
Sore throat	–0.32 ± 0.57	–0.17 ± 0.45	0.1333
Hoarseness	–0.23 ± 0.43	–0.09 ± 0.38	0.0314*
Feeling of a “knot” in the throat	–1.50 ± 0.91	–0.88 ± 0.97	0.00053*
Desire to clear the voice	–0.27 ± 0.55	–0.12 ± 0.43	0.0974
Persistent cough that worsens with the supine position	–0.50 ± 0.67	0.24 ± 0.54	0.0340*
Early satiety	–1.82 ± 0.66	–1.46 ± 0.65	0.0258*
Feeling of fullness after meals	–2.09 ± 0.81	–2.20 ± 0.70	0.7453
Burning/epigastric pain	–2.18 ± 0.66	–2.28 ± 0.70	0.5759

Mean change (T3 vs. baseline) severity scores. *P < 0.05, MD + PPI vs. MD alone (Wilcoxon rank-sum test); exploratory analysis (n = 22 vs. 133). No multiplicity adjustment. MD: medical device; PPI: proton pump inhibitor.

**Table 6:** Change in Symptom Frequency Score From Baseline to T3 in the MD Only and MD + PPI Groups

Symptom	Variations at T3 vs. baseline (mean ± SD)/MD ± PPI	Variations at T3 vs. baseline (mean ± SD)/MD only	P value
Pyrosis	–1.82 ± 0.91	–1.47 ± 0.76	0.1431
Acid regurgitation	–2.23 ± 0.92	–2.22 ± 0.85	0.8634
Epigastric pain	–2.23 ± 0.81	–2.18 ± 0.78	0.5846
Sore throat	–0.64 ± 0.85	–0.19 ± 0.60	0.0014*
Hoarseness	–0.32 ± 0.57	–0.15 ± 0.53	0.0804
Feeling of a “knot” in the throat	–1.50 ± 1.01	–0.95 ± 1.10	0.0239*
Desire to clear the voice	–0.14 ± 0.35	–0.17 ± 0.58	0.8624
Persistent cough that worsens with the supine position	–0.55 ± 0.67	–0.23 ± 0.58	0.0085*
Early satiety	–1.73 ± 0.94	–1.41 ± 0.76	0.0695
Feeling of fullness after meals	–1.86 ± 0.71	–2.03 ± 0.83	0.3726
Burning/epigastric pain	–2.23 ± 0.87	–2.11 ± 0.80	0.4760

Mean change (T3 vs. baseline) frequency scores. *P < 0.05, MD + PPI vs. MD alone (Wilcoxon rank-sum test); exploratory analysis (n = 22 vs. 133). No multiplicity adjustment. MD: medical device; PPI: proton pump inhibitor.

## Discussion

This real-world survey provides insights into the experience of patients using Piromal^®^ for upper gastrointestinal symptoms commonly reported in routine clinical practice. Participants reported a high burden of typical symptoms before the treatment, particularly heartburn and postprandial fullness, which were also the main causes of nighttime discomfort. Sleep disturbances were common, and the impact on daily and social activities confirmed the significant impact of GERD-related symptoms on QoL. During the observation period, the survey consistently showed improvements in the severity and frequency of symptoms. Reductions were observed for typical, atypical, and dyspeptic symptoms, as well as for nocturnal disturbances.

Although the study design does not allow causal inference, the temporal association between product use and symptom improvement suggests that the MD may support symptom management as reflected by improvements in PROs, and this trend is further strengthened by improvements in QoL indicators [20]. At the same time, because the questionnaire was not specifically designed to distinguish GERD from functional upper gastrointestinal disorders, some of the observed improvements may also reflect changes in overlapping nonspecific symptom domains rather than exclusively GERD-related manifestations. Accordingly, the present findings are more appropriately interpreted as improvements in PROs than as evidence of a specific effect on the underlying cause of symptoms.

The analysis of perceived symptom improvement showed that participants reported high levels of subjective benefit for typical and dyspeptic symptoms. Atypical symptoms experienced a less significant perceived improvement, which is in line with their lower degree of severity at baseline. This pattern aligns with the known clinical behavior of extra-esophageal manifestations, which often fluctuate and may respond more slowly to protective or barrier-based interventions [21].

Early benefits were already evident at 7 and 15 days, indicating a progressive pattern of symptom relief over the short follow-up period. In addition, the survey showed an overall favorable acceptability profile, with high ratings for palatability, tolerability, ease of use, and satisfaction. At the same time, nausea was reported by 23.9% of participants and flatulence by 14.8%, making these the most frequently reported treatment-emergent events. This finding deserves explicit consideration. Because these events were collected as dichotomous PROs in an anonymous exploratory survey, without baseline assessment, severity grading, or systematic collection of potential confounders such as concomitant medications, dietary changes, smoking, or other lifestyle factors, they cannot be clearly attributed to the MD. Accordingly, although the overall acceptability of the product remained satisfactory, the observed nausea signal should be interpreted cautiously and warrants further evaluation.

Successful treatment of GERD requires effective symptom management, and accurate assessment of symptom patterns is essential to prevent long-term complications [22]. The survey findings suggest that the MD was associated with reductions in the severity and frequency of both diurnal and nocturnal symptoms, which may support more effective symptom control in routine practice. The observed reduction in the impact of symptoms on daily and social activities further indicates a potential benefit for patients’ QoL [23].

The subgroup analysis showed differences in specific symptoms between MD alone versus MD combined with a PPI. Participants using the combination reported greater reductions in certain typical and atypical symptoms, as well as in nocturnal disturbances. In patients with a greater persistence of these specific symptoms, the results may suggest a complementary effect when MD is combined with PPIs. However, because the combination subgroup was small, non-randomized, and potentially affected by baseline confounding, these findings should be considered exploratory only. At the same time, for the most symptom domains, no consistent superiority of combination therapy emerged, and symptom improvement and favorable PROs were also observed in the MD-alone group. This pattern may suggest that the MD itself contributes meaningfully to symptom relief in some patients even without concomitant PPI use, although the present data do not allow firm clinical conclusions and do not provide evidence regarding PPI dose reduction or discontinuation.

PPIs remain the most effective pharmacological therapy for GERD, and their benefits generally outweigh potential risks when used appropriately [24, 25]. However, inappropriate long-term prescribing—related to excessive duration, high doses, or lack of clear indication—has been widely documented, particularly in Western countries [26–29]. Patients with comorbidities, polypharmacy, or risk factors for long-term adverse effects require careful monitoring. Current guidelines recommend using PPIs only when clearly indicated, at the lowest effective dose, and with periodic reassessment to determine whether therapy can be tapered or discontinued [24, 26, 30].

Given the growing attention to deprescribing strategies in GERD management, the present findings should not be interpreted as evidence supporting a deprescribing effect of the MD. Rather, they may justify future controlled studies designed to evaluate whether mucosal protectants could have a supportive role within structured PPI step-down or discontinuation strategies.

### Limitations of the study

Despite the encouraging results, this study has several limitations. The survey relied on PROs, which may be influenced by subjective perception and recall bias. The absence of a control group prevents distinguishing the effect of the MD from the natural variability of symptoms. The sample, although substantial, may not fully represent the broader GERD population, and unmonitored factors such as diet, lifestyle, or concomitant medications may have influenced outcomes. Finally, the 25-day observation period does not allow assessment of long-term symptom control or sustained benefit.

A further limitation of the present study is that the questionnaire was developed specifically for this survey and was not formally validated. Therefore, the PROs should be considered exploratory and hypothesis-generating rather than a psychometrically validated assessment of disease severity or treatment response. In addition, although participants had received a formal clinical diagnosis of GERD from their treating specialists, the tool was designed to identify reflux-related symptom patterns in routine clinical practice and was not specifically developed to distinguish GERD from functional upper gastrointestinal disorders or to characterize functional symptom components. Consequently, although some symptom domains assessed in the survey may overlap with functional manifestations, the present data do not allow firm conclusions regarding a specific effect of the MD on functional symptoms. Accordingly, the potential contribution of the MD to functional upper gastrointestinal symptoms remains conjectural and requires additional investigations with a more refined phenotyping of the study population. In addition, due to the anonymous and exploratory nature of the survey, potentially relevant confounders such as dietary shifts, weight variation, smoking, and alcohol consumption could not be systematically collected and may have influenced the observed outcomes.

Moreover, given the anonymous and exploratory nature of the survey, although the GERD diagnosis had been provided by the specialist, objective confirmation was not available for all participants. As a result, possible functional gastrointestinal disorders could not be formally excluded. Finally, in line with the PROs literature, where exploratory real-world analyses often prioritize descriptive characterization of symptom patterns over formal confirmatory testing, the observed P values should be interpreted as nominal and hypothesis-generating rather than confirmatory. Given the large number of symptom-domain comparisons and the absence of formal correction for multiple testing, the risk of type I error cannot be excluded. Similarly, the subgroup analyses comparing MD alone and MD + PPI were based on a small, non-randomized subgroup and should therefore be considered exploratory only, without implying definitive between-group conclusions.

### Conclusions

This real-world survey suggests that the MD may improve PROs and QoL indicators over 25 days of use, with improvements observed across typical, atypical, and dyspeptic symptom domains reported by patients with reflux-related symptoms. Overall, the MD was well tolerated, with high patient satisfaction, despite the observed nausea signal, which warrants cautious interpretation and further assessment. Although the observational design does not allow causal inference, the temporal association between product use and symptom improvement highlights its potential role as a supportive option in routine practice. Symptom improvement was reported both in patients using the MD alone and in those using it alongside PPIs, with exploratory subgroup findings suggesting a possible complementary effect in some symptom domains, but without a consistent advantage of combination therapy across most outcomes. These findings also suggest that favorable PROs may occur with the MD alone in a substantial proportion of patients. Given the current focus on appropriate PPI use and deprescribing strategies, these exploratory findings generate a hypothesis for future controlled studies evaluating whether mucosal protectants may support patients during PPI dose reduction or discontinuation. However, because of the observational design, the anonymous and exploratory nature of the survey, the lack of objective confirmation of GERD for all participants, the results should be interpreted as hypothesis-generating rather than confirmatory.

Further controlled studies with longer follow-up, more refined phenotyping of symptom etiology, and prospective assessment of concomitant therapies are needed to better define the role of the MD in the management of upper gastrointestinal symptoms and to clarify whether it may have a supportive role in structured PPI management pathways.

## Data Availability

The anonymized datasets generated and analyzed during the current study are not publicly available due to institutional policy but are available from the corresponding author on reasonable request.

## Abbreviations

GERD: gastroesophageal reflux disease
PROs: patient-reported outcomes
MD: medical device
PPIs: proton pump inhibitors
QoL: quality of life
VNRS: Verbal Numeric Rating Scale
EU: European Union
GDPR: General Data Protection Regulation
SAS: Statistical Analysis System
GERD-Q: Gastroesophageal Reflux Disease Questionnaire
GSAS: Gastroesophageal Symptom Assessment Scale
RDQ: Reflux Disease Questionnaire
